# PD-1: Its Discovery, Involvement in Cancer Immunotherapy, and Beyond

**DOI:** 10.3390/cells9061376

**Published:** 2020-06-01

**Authors:** Yasumasa Ishida

**Affiliations:** Division of Biological Science, Nara Institute of Science and Technology, 8916-5 Takayama-cho, Ikoma-shi, Nara 630-0192, Japan; ishiday@bs.naist.jp; Tel.: +81-743-72-5531

**Keywords:** PD-1, T cell, subtractive hybridization, self-nonself discrimination, cancer, immunotherapy

## Abstract

On December 10, 2018, I was sitting among the big crowd of audience, as one of the invited guests to the ceremony, in the Stockholm Concert Hall. When King of Sweden Carl XVI Gustaf bestowed the diploma and medal of Nobel Prize of Physiology or Medicine 2018 on Dr. Tasuku Honjo and shook his hand for a while, surrounded by the thunderous applause and energetically blessing orchestral music, I thought that it had been a long journey for the molecule that we had first isolated in the early 1990s. Although it was truly a commemorable moment in the history of the programmed death-1 (PD-1) research, I believe we still have a long way to go. In this review article, I will explain why I think so, particularly by focusing on the potential role(s) that PD-1 appears to play in self-nonself discrimination by the immune system.

## 1. Introduction

In the early 1990s, a novel gene was discovered in Kyoto University, Japan, in search for the molecular mechanisms involved in self-nonself discrimination by the immune system [1]. In 1989, a UK team showed that self-reactive (potentially harmful) immature T lymphocytes (T cells) in the thymus undergo programmed cell death (apoptosis) [2]. This observation led the Kyoto University researches to assume that, if they are able to discover the genes strongly associated with the deaths of immature T cells, some of them would become good candidates for the key molecules playing pivotal roles in self-nonself discrimination. Only one gene was discovered at that time in a challenging screening experiment in molecular biology, and the gene (or its product) was named programmed death-1 (PD-1), with a hope that it would be somehow involved in the apoptosis-inducing processes of self-reactive immature T cells [1]. However, it turned out, several years later, that the novel molecule had nothing to do with the induction of programmed cell death/apoptosis [3,4,5,6]. Very interestingly, the wonderful developments in cancer immunotherapy in the recent years strongly suggest that, while PD-1, induced to be expressed on the surface of activated T cells, forces T cells to ignore cancer cells as one of the authentic ‘self’ components (i.e., prevents T cells from attacking cancer cells), the antibody-mediated blockade of PD-1′s function makes activated T cells aware of the ‘nonself’ nature of cancer cells and unleashes their cytotoxicity. Therefore, although PD-1 was not directly involved in the cell death-inducing processes (despite its ominous name), it could still be playing crucial roles in self-nonself discrimination, as initially expected in the early 1990s.

## 2. Historical Background

By the end of the 1970s, critical parts of the human and mouse immunoglobulin (Ig) genes had already been identified, and the genetic basis for the enormous diversification of the variable regions of antibody molecules had been elucidated [7,8]. In the early 1980s, on the other hand, there was a fierce competition among immunologists for the discovery of the molecular nature of T-cell antigen receptors (TCRs). Three groups, led by Dr. Ellis L. Reinherz of Dana-Farber Cancer Institute in Boston, Drs. John Kappler and Philippa Marrack of National Jewish Hospital and Research Center in Denver, and Dr. James P. Allison of the University of Texas System Cancer Center Science Park in Texas, took a similar strategy of affinity purification of the TCR proteins by using anti-clonotypic monoclonal antibodies (that recognized the hypervariable regions of the TCR molecules) and showed remarkable successes [9,10,11]. Many thought at that time (in the middle of 1983) that either of the above three groups in the United States would win the TCR-cloning race.

In March 1984, three Nature Articles were published by the groups of Dr. Tak W. Mak of University of Toronto, Canada, and Dr. Mark M. Davis of Stanford University, USA [12,13,14]. There, they reported the discovery of the TCR genes by using the extremely elegant subtractive (or differential) hybridization technique in molecular biology. Both of the groups were not professionals of protein purification but were able to discover the long-sought molecules only with their well-controlled experiments at the DNA-RNA level. Many immunologists, especially the young ones all over the world, were deeply inspired by the great achievements and strongly wished to perform similar subtractive hybridization experiments in their own careers in the future.

One of the desperate losers in this TCR-cloning race was Dr. James P. Allison, who 34 years later shared the Nobel Prize in Physiology or Medicine 2018 with Dr. Tasuku Honjo for their discovery of the novel type of cancer immunotherapy.

## 3. Self-Nonself Discrimination and T-Cell Deaths

In the late 1980s, bioscience researchers were beginning to understand an attractive and novel notion of apoptosis and/or programmed cell death. In 1989, a UK team published a Nature paper [2], showing that, when strongly stimulated through their TCRs, developing immature T cells in the thymus immediately realize that they are harmfully reacting against self-components and commit suicide by undergoing apoptosis/programmed cell death.

Dr. Jonathan D. Ashwell and colleagues at National Institutes of Health (NIH), USA, published an interesting paper indicating that mouse T-cell hybridomas produced by the cell fusion between a thymoma cell line BW5147 and a normal mouse peripheral T cell can be induced to die in vitro via strong stimulation towards their TCRs [15]. Their behavior after the TCR stimulation appeared to be very similar to that of self-reactive immature T cells in the thymus [2]. More interestingly, they also showed that such activation-induced death of mouse T-cell hybridomas depended upon de novo gene expression [16].

When the researchers in the Honjo laboratory in Kyoto University, Japan, noticed these interesting papers in 1989, they realized that they might be able to achieve their target by performing the state-of-the-art molecular-biology experiments: if they are able to isolate the genes strongly associated with the apoptosis/programmed death of self-reactive (strongly stimulated) immature T cells (or T-cell hybridomas) by using the subtractive-hybridization technique, such genes must become the very important clues for them to initiate their investigation on the molecular mechanisms involved in self-nonself discrimination.

At that time, however, the members in the Honjo laboratory were afraid that subtraction in just one direction (i.e., ‘stimulated T-cell hybridoma’ minus ‘unstimulated T-cell hybridoma’) would not be able to reduce the number of isolated cDNA clones to the practical ones because such simple subtraction should also enrich for the genes that are involved in typical T-cell activation events (like the cDNA for interleukin-2 (IL-2)). Therefore, they decided to incorporate into their subtractive hybridization experiments another type of cell-death induction system in vitro: apoptosis was induced in a mouse hematopoietic progenitor cell line by the deprivation of its growth factor interleukin-3 (IL-3), and they confirmed that the death induction of that cell line was also dependent upon de novo gene expression. If it is possible for them to isolate the gene(s) in which expression is commonly upregulated in two entirely distinct cell lines that are stimulated to undergo apoptosis in different ways, they thought that the chance of discovering the gene(s) deeply involved in the apoptosis-inducing processes would be reasonably high.

## 4. Cell-Death Research in the Early 1990s

Here, I need to briefly review the situation around the cell-death research at that time. The importance of the genetically regulated cell deaths was beginning to be appreciated among bioscience researchers, and Dr. H. Robert Horvitz’s team in Massachusetts Institute of Technology in Cambridge, USA, was leading the field. By 1986, they had already identified two genes, ced-3 and ced-4, that are required for the physiological cell deaths taking place during the development of a nematode Caenorhabditis elegans (C. elegans) [17].

In the research field of humans or mice, on the other hand, an Australian group led by Drs. Suzanne Cory and Jerry M. Adams of the Walter and Eliza Hall Institute of Medical Research discovered that Bcl-2, which was first identified from a chromosomal breakpoint in human follicular B lymphoma cells by Drs. Yoshihide Tsujimoto and Carlo M. Croce of the Wistar Institute in Philadelphia, USA [18], is able to suppress cell deaths without causing cell proliferation [19]. It was in 1992 that the ced-9 gene product that negatively regulates programmed cell deaths during the development of C. elegans was shown to possess functional and structural similarities to mammalian Bcl-2 [20].

In 1993, the first member of a group of cysteine proteases (that were later given with the name ‘caspases’) was reported as a functional and structural mammalian homolog of the nematode cell-death gene ced-3 product [21]. For the discovery of Apaf-1, the mammalian homolog of the nematode ced-4 gene product, it took several more years [22]. In summary, it would be important here to note that nothing had been elucidated as crucial molecular mechanisms involved in mammalian programmed cell deaths before 1990–1991, when the researches initiated their subtractive hybridization experiments in Dr. Honjo’s laboratory in Kyoto University.

## 5. Discovery of PD-1

In 1991, Dr. Honjo’s group decided to initiate subtractive hybridization in which two independent apoptosis-induction systems (i.e., the strong TCR stimulation of mouse T-cell hybridomas and the growth-factor deprivation from mouse hematopoietic progenitor cells) were combined. They thought that it might be possible for them to discover the gene(s) deeply involved in induction of programmed deaths of mammalian cells, as well as those playing crucial roles in self-nonself discrimination by the immune system [23].

Dr. Yusuke Yanagi, who discovered a gene for human TCR in Dr. Tak W. Mak’s laboratory in 1984 [12], provided Dr. Honjo’s team with his detailed protocol of subtractive hybridization. During the summer of 1991, the researchers in the Honjo group performed many preliminary control experiments in subtractive hybridization. They initiated the ‘two-dimensional’ (or combined) subtractive hybridization experiments in the middle of August and, by the end of September, were able to isolate four independent cDNA clones that they initially believed to have been derived from four different candidate genes. Very interestingly, however, the reality was that all of the four cDNA clones were originated from the same single gene, which they later named programmed death-1 (PD-1) in the middle of October 1991, hoping that the novel gene could have something to do with apoptosis/programmed death of immature T cells. Next year (in 1992), they published their first PD-1 paper [1].

## 6. The PD-1 Research in the Honjo Laboratory in the Late 1990s

In the late 1990s, Dr. Honjo’s group at Kyoto University elucidated the basic function(s) of the newly identified molecule. PD-1, one of the type I trans-membrane proteins, transiently expressed on the cell surface of activated T cells (and also B cells), was a receptor that receives the negative regulatory signals from the surrounding cells and sends such signals into the cytoplasm of activated T cells [3,4,5,6]. In other words, Dr. Honjo’s group showed that: (1) From the functional point of view, PD-1 is not directly involved in the induction of apoptosis/programmed death of immature T cells. (2) Instead, PD-1 is a negative regulator of the immune responses of T cells.

Because of this, many people (including Dr. Honjo) say that PD-1 was discovered just coincidentally. Exactly, PD-1 was not a molecule directly involved in the apoptosis-inducing processes nor an ultimate negative regulator of T-cell survival. Instead, PD-1 was a novel type of the negative regulator on T-cell responses. Therefore, we might be able to say that PD-1 was not so distantly located from the original mark. In addition, the goal of Dr. Honjo’s group at that time was to elucidate the molecular mechanisms involved in self-nonself discrimination by the immune system. Today, no one believes that PD-1 is an apoptosis-inducing gene. At the same time, however, no one doubts that PD-1 plays some kind of pivotal roles in self-nonself discrimination by T cells (as described below).

Some of the graduate students in the laboratories of Drs. Nagahiro Minato and Tasuku Honjo also discovered the potential usefulness of the blockade of the PD-1 signaling pathway in cancer therapy [24]. An ally of a group in the Ono pharmaceutical company (Osaka, Japan) led by Dr. Shiro Shibayama, who also had a training in Dr. Honjo’s laboratory in the early 1990s, and another led by Drs. Alan J. Korman and Nils Lonberg in Medarex/Bristol-Myers Squibb (USA) succeeded in the development of a fully human anti-human PD-1 monoclonal blocking antibody of the IgG4 subclass (nivolumab/Opdivo).

## 7. Recent Developments in Cancer Immunotherapy

Recently, the immunotherapies using anti-CTLA-4 and/or anti-PD-1 monoclonal blocking antibodies have revolutionized the field of human cancer treatment [25,26,27]. For instance, several anti-PD-1 and anti-PD-L1 monoclonal blocking antibodies, including nivolumab, pembrolizumab, and atezolizumab, have been approved by Food and Drug Administration (FDA) of the United States as an effective drug for the treatment of malignant melanoma, non-small cell lung cancer (NSCLC), renal cell carcinoma (RCC), Hodgkin lymphoma, squamous cell carcinoma of the head and neck, hepatocellular carcinoma, and others.

Consequently, Drs. James P. Allison and Tasuku Honjo have been frequently honored with distinguished prizes and awards, including the Tang Prize (Taiwan), the William B. Coley Award (USA), the Lasker Award (USA), and the Nobel Prize (Sweden), for their significant contribution to cancer immunotherapy.

Both CTLA-4 and PD-1 are the negative regulators of immune responses, belonging to the same CD28 family. In addition, both CTLA-4 and PD-1 are currently being used as the targets of antibody-mediated functional blockade in human cancer immunotherapy. Historically speaking, Dr. James P. Allison first showed that the CTLA-4 blockade is effective in the murine model of cancer immunotherapy [28]. Drs. Tasuku Honjo and Nagahiro Minato were able to prove the same thing for PD-1 about six years later [24]. This is why many people think that Dr. James P. Allison was the person who first opened the door of current cancer immunotherapy and why the Lasker award 2015 in the field of clinical medicine went to him alone.

However, the reality of cancer immunotherapy at present in clinics all over the world is another story. Side effects of cancer immunotherapy using the CTLA-4 antibody (ipilimumab) are much more serious and severe than those using the PD-1 antibodies. Although the FDA approval was earlier for CTLA-4 than for PD-1, the majority of clinicians right now believe that the CTLA-4 blockade would not be able to survive as a mono-therapy: it would be utilized only in combination with some other things, including the PD-1 blockade. On the other hand, the list of cancer types that can be treated through the PD-1 blockade is still constantly expanding. Basically, the PD-1 blockade is safer and more effective than the CTLA-4 blockade.

To understand the differential effects between the CTLA-4 blockade and the PD-1 blockade in cancer immunotherapy, we first need to think about the physiological functions of CTLA-4 and PD-1. First of all, the phenotypes of the knockout mice for these two molecules are dramatically different. All of the CTLA-4 knockout mice die of the extremely severe autoimmune diseases within three weeks after birth [29,30]. In contrast, the PD-1 knockout mice (especially on the C57BL/6 genetic background) are perfectly healthy for about one year after birth [4]. Sporadic and mild autoimmune diseases gradually begin to arise in the aged PD-1 knockouts, only about one year after birth.

This is because CTLA-4 is negatively regulating virtually all immune reactions, while PD-1 is suppressing only a limited type of immune responses, like those against cancer cells. Consequently, in the CTLA-4 knockout mice, all immune reactions become unleashed and uncontrolled, while, in the PD-1 knockout mice, only a certain type of immune responses are gradually re-activated [4,29,30]. After blocking the functions of CTLA-4 or PD-1 by using antibodies, the same patterns of things happen in cancer patients: serious and grave side effects with the CTLA-4 antibody; and only mild adverse events with the PD-1 antibody [25,26,27].

Recent findings also suggest that many of the anti-CTLA-4 antibodies with the anti-tumor activity, including ipilimumab (a fully human anti-human CTLA-4 monoclonal antibody of the IgG1 subclass), might not be acting simply as the checkpoint blocker. Instead, such antibodies could also be unleashing a broad spectrum of immune reactions by depleting regulatory T cells (Tregs), which are well-known to express the CTLA-4 molecules on their cell surface constitutively, through their antibody-dependent cellular cytotoxicity (ADCC) activity [31,32,33]. In the experimental mouse studies in which different anti-mouse CTLA-4 monoclonal IgG antibodies (either natural or recombinant) with distinct Fc structures were compared, those with higher affinity to Fc-gamma receptors (FcγRs) showed the stronger Treg-depleting and anti-tumor activities [31,32]. In addition, the anti-tumor activity was completely abolished in the anti-CTLA-4 antibodies without the FcγR-binding capability [32]. In the human immune system that matters for the clinical medicine, the ADCC activity of IgG1 (e.g., ipilimumab) is much higher than that of IgG4 (e.g., nivolumab). Taken together, these numerous findings in recent years strongly suggest that the main effect of anti-CTLA-4 monoclonal antibodies must be mediated by the depletion of Tregs inside tumors, not by the checkpoint blockade.

## 8. Remaining Questions and a Hypothesis about the ‘Real’ Physiological Function(s) of PD-1

As described above, the PD-1 knockout mice (especially on the C57BL/6 genetic background) are perfectly healthy, at least for about one year after birth [4]. In some of the aged PD-1 knockout mice (that are older than one year after birth), however, we can see the signs of mild glomerulonephritis and arthritis [4]. Then, what is PD-1 doing in young mice? In addition, why does such mild and sporadic autoimmunity arise only in aged mice?

Upon blockade of the PD-1′s function(s) in the body of some cancer-bearing animals or patients by using the inhibitory antibodies, the killer T cells begin to attack cancer cells. Dr. Robert D. Schreiber’s group in Washington University in St. Louis, MO, USA, showed in their chemically induced sarcoma model in mice that such killer T cells activated after the PD-1 blockade recognized mutated peptide antigens (so called ‘neoantigens’) derived from the chemically mutagenized genome of the sarcoma cell line in the context of the major histocompatibility complex (MHC) molecules [34]. This result is particularly interesting because it strongly suggests that, although killer T cells are capable of recognizing and responding to the neoantigens expressed on cancer cells, PD-1 usually halts such beneficial immune reactions. Then, what is PD-1 protecting there? In the normal condition (i.e., without injection of the inhibitory anti-PD-1 antibodies), PD-1 appears to be protecting cancer cells from the attack by the immune system. Is PD-1 on our side? Or on the side of cancer cells?

Immunologists usually say that PD-1 is involved in the maintenance of peripheral tolerance. These words give us some kind of impression about the immune reactions, and many of us might be satisfied with it. Unfortunately, however, the image here still looks very vague and ambiguous. We have to precisely understand what kind of immune reactions can be, or cannot be, suppressed by PD-1 because PD-1 is not an all-round player in the negative regulation of immune reactions [35].

**Figure 1:**
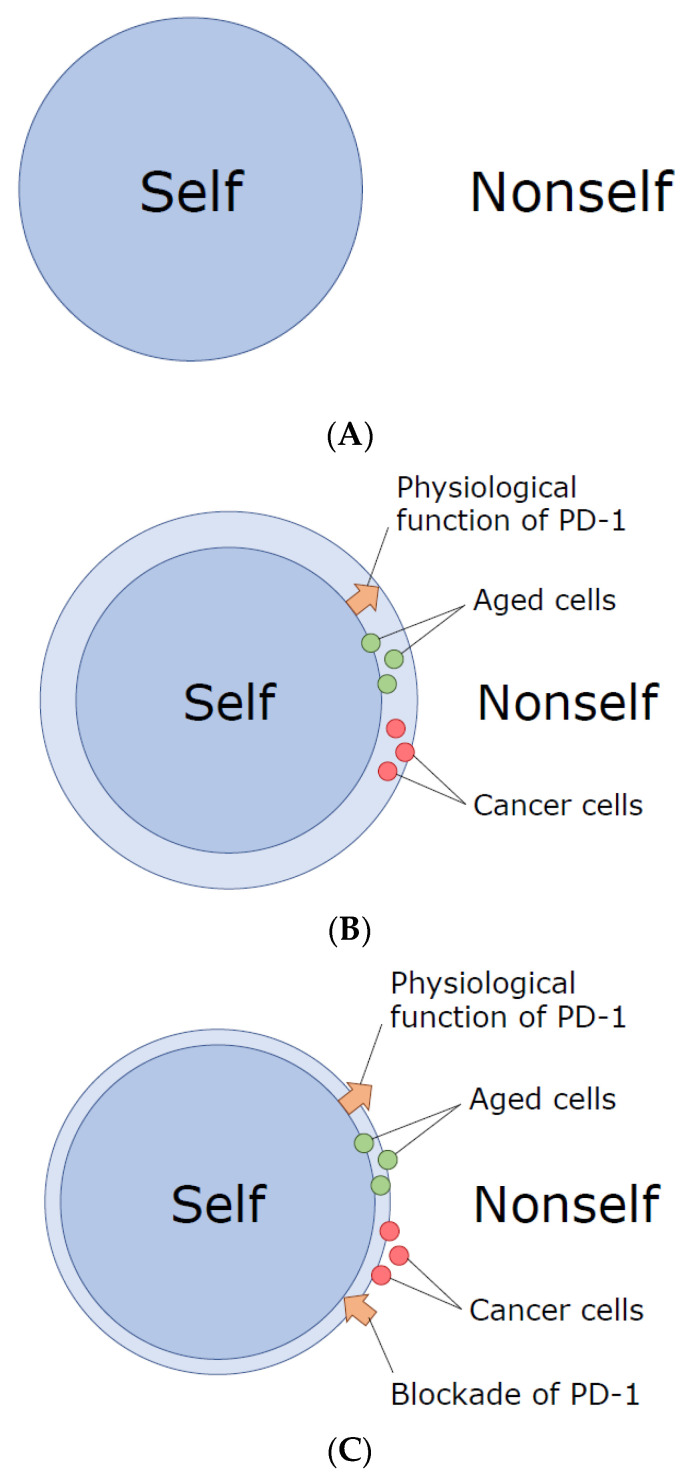
A hypothesis about the physiological function of programmed death-1 (PD-1): Demarcation of the border between ‘slightly altered self’ and ‘nonself’. (**A**) Originally, when we are young, the border between ‘self’ and ‘nonself’ is obvious. (**B**) However, subtle changes gradually accumulate as cells age, and the self-nonself border becomes ambiguous. In order to avoid the attacking against ‘slightly altered’ aged somatic cells by the exquisitely evolved immune system of higher vertebrates, we obtained PD-1. Here, PD-1 functions to widen and re-define the border between ‘slightly altered self’ in aged individuals and ‘nonself’. Cancer cells are clever enough to sneak into this chink between the original self and the slightly altered self, so that they can be protected by PD-1 and avoid the immune-cell attack. (**C**) When we block the activity of PD-1, the self-nonself border moves back (closer) to the original one, and a fraction of cancer cells (and also aged somatic cells) get re-defined as nonself, allowing the highly evolved immune system to attack them.

We might be able to hypothesize as follows. When we are very young, cells in our body are pretty intact, and the immune system can easily distinguish ‘self’ from ‘nonself’ (Figure 1A). As we age, however, tiny alterations are gradually accumulated in a variety of components of our cells, and our highly evolved immune system with superb sensitivity and specificity, equipped with Variable (Diversity) Joining (V(D)J) recombination, somatic hypermutation, and so on, could detect and misrecognize such small alterations (‘slightly altered self’) as ‘nonself’, initiating the battles against them. In my personal view, we appear to have acquired PD-1 during evolution so that we can avoid (or suppress) autoimmunity against the gradually accumulated small changes in our cellular components (Figure 1B). In other words, we can be tolerant to the ‘slightly altered self’, especially in aged individuals, due to the presence of PD-1.

As described before, young PD-1 knockout mice do not show any particular immunological abnormalities. This is probably because they still have not accumulated small alterations in their cellular components. Aged PD-1 knockout mice, on the other hand, have accumulated a significant amount of such changes in their cells, and in the absence of PD-1, our immune system misrecognizes them as ‘nonself’ and initiates the attacks against them.

In the genome of cancer cells (particularly in those of aged individuals), numerous somatic mutations are usually accumulated. While only a limited fraction of them are classified as the driver mutation that could provide a selective growth advantage and thus promote cancer development, the vast majority are the neutral passenger mutations that have nothing to do with the cancer initiation and/or promotion [36,37]. Some of such mutations in the genome, either the driver ones or passenger ones, could result in the generation of neoantigens, a portion of which might be able to be presented on the cell surface and recognized by T cells as the slightly altered self components [34,38]. However, when PD-1 halts harmful immune reactions against slightly altered normal somatic cells especially in aged individuals, the beneficial responses toward neoantigens generated in cancer cells also appear to be suppressed by PD-1 (Figure 1B).

In a good contrast, PD-1 is not capable of negatively regulating (nor suppressing) the strong immune reactions against the apparent ‘nonself’ (e.g., microorganisms that cause acute infections in animals like the influenza or measles viruses). Immunity against such apparent ‘nonself’ can easily and perfectly be provoked even in the presence of PD-1 in part because one of the ligands of PD-1, PD-L1, is sequestered and functionally inactivated in cis by the CD80 (B7-1) molecules the expression of which is strongly upregulated on antigen-presenting cells upon inflammation [35].

Upon cancer immunotherapy with anti-PD-1 or PD-L1 monoclonal antibodies, immune reactions against the slightly altered self-components could be generally re-activated or unleashed. This is probably why cancer patients with checkpoint-blockade immunotherapy sometimes experience so-called immune-related adverse events (irAE) [25,26,27]. Importantly, however, the number of somatic mutations in the genome is usually much larger in cancer cells than in normal somatic cells, and, from the immunological point of view, a fraction of cancer cells are more distantly located than normal somatic cells of aged individuals from the original ‘self’. Therefore, we should usually be able to observe some degree of selectivity in the PD-1-mediated cancer immunotherapy, having the stronger immune reaction against cancer cells than toward normal somatic cells (Figure 1C).

With the final proof of the above hypothesis, we would be more firmly convinced that PD-1 plays the extremely important role(s) in self-nonself discrimination.
